# *Burkholderia pseudomallei* hybrid assemblies of 20 environmental isolates from Vietnam

**DOI:** 10.1128/mra.00988-25

**Published:** 2026-02-27

**Authors:** Sarah Weigl, Johanna Dabernig-Heinz, Trung Thanh Trinh, Ivo Steinmetz, Gabriel E. Wagner, Sabine Lichtenegger

**Affiliations:** 1 Diagnostic and Research Institute of Hygiene, Microbiology and Environmental Medicine, Diagnostic and Research Center for Molecular Biomedicine, Medical University of Graz, Graz, Austria; 2 VNU-Institute of Microbiology and Biotechnology, Vietnam National University, Hanoi, Vietnam; University of Southern California, Los Angeles, California, USA

**Keywords:** *Burkholderia pseudomallei*, hybrid genome, Vietnam, soil, nanopore, autocycler

## Abstract

*Burkholderia pseudomallei* is a soil-dwelling bacterium causing melioidosis, a severe and often fatal disease (10–40% lethality). Here, we report 20 complete high-quality, closed genomes (7.0–7.5 Mbp, ~68.1% GC) from Vietnamese soil isolates, assembled from Oxford Nanopore and Illumina sequencing data.

## ANNOUNCEMENT

The bacterium *Burkholderia pseudomallei* is endemic in tropical and subtropical areas, where it is commonly found in moist soils and water. *B. pseudomallei* is the causative agent of melioidosis, a severe infectious disease with a high mortality rate (1–4). Common infection routes include environmental exposure via percutaneous inoculation, inhalation, and ingestion (1).

By combining data from Oxford Nanopore and Illumina sequencing, we generated complete, high-quality genomes of 20 agricultural soil isolates from Chau Dinh commune, Quy Hop district, Nghe An province in Vietnam (5)*,* representing a significant contribution to the available genomic resources. To our knowledge, only one closed Vietnamese *B. pseudomallei* genome had been deposited prior to this study. Therefore, the genomes provided here represent a valuable resource for understanding regional diversity and bacterial evolution.

For isolation of bacteria, 10 g of soil was enriched in 20 mL threonine-basal salt solution with colistin (TBSS-C50) (6) for 4 days at 40°C. Subsequently, 1 mL was transferred to 9 mL TBSS-C50 with erythritol as the sole carbon source (7) and incubated as before. Subculturing was performed on Ashdown agar to isolate single colonies, and TTSS-1 PCR was performed for species identification according to Trinh et al. and Novak et al. (7, 8).

For DNA extraction, a full 1 µL inoculation loop of well-isolated colonies of each strain was picked from Columbia agar containing 5% sheep blood (BD Biosciences; 37°C for 24 h). DNA was extracted using the NucleoSpin microbial DNA kit (Macherey-Nagel) with slight modifications (9) and cleaned with AMPure XP bead solution (Beckman Coulter) (9). DNA was quantified using a Qubit 4 fluorometer with Qubit BR assay kit (Thermo Fisher Scientific), yielding about 15–20 µg DNA per extraction. DNA extractions for Illumina and Nanopore sequencing were performed independently from the same cryo-stock originating from one single colony.

For Nanopore sequencing, 400 ng DNA was used with the Native Barcoding Kit 24 V14 (Q20+) (SQK-NBD114.24) for library preparation according to the manufacturer’s instructions with R10.4.1 flow cells (Oxford Nanopore Technologies) without size selection. Dorado (v0.7.2; model “dna_r10.4.1_e8.2_400bps_sup@5.0.0”) was used for basecalling, demultiplexing, and adapter trimming. Read quality was assessed with Nanoplot (v1.44.1). For Illumina, the Nextera XT library preparation kit was used with an Illumina MiSeq instrument and a MiSeq reagent kit version 2 (Illumina, Inc.) for 2 × 100 bp or 2 × 150 bp paired-end sequencing. Read quality was assessed with FastQC (v0.12.1).

Autocycler (v0.3.0.) (10) was used for long-read assembly with standard settings (with Flye [v.2.9.5] [11], Minasm [v.0.3] with minimap2 [v2.28] [12], NextDenovo [v.2.5.2] [13], Raven [v.1.8.3] [14], and NECAT [v.0.0.1_update20200803]) (15). Only isolates with closed, circular assemblies were included. Assemblies were polished with Medaka (v1.11.3; model “r103_min_high_g360”) (16). Illumina reads were used to generate hybrid assemblies through polishing by applying Fastp (v0.24.1), Polypolish (v0.6.0), and Pypolca (v0.3.1; careful mode) (17). Quast (v5.2.0) was used for quality assessment and annotation was performed through the NCBI Prokaryotic Genome Annotation Pipeline (PGAP v6.10) (18). Default parameters were used except where otherwise noted.

**Fig 1:**
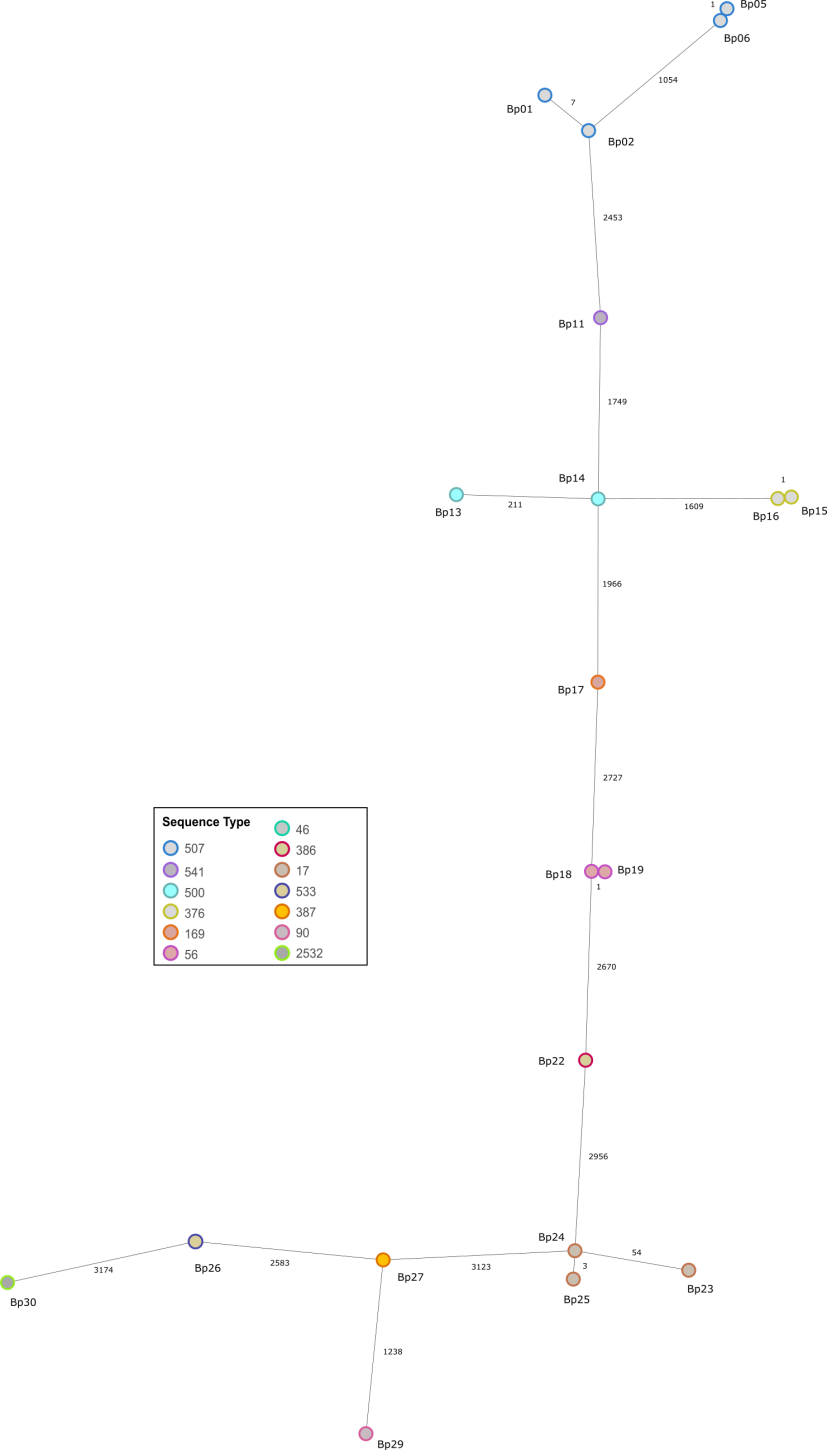
cgMLST typing of 20 *B. pseudomallei* strains from Vietnam was conducted as previously described in Lichtenegger et al. and Weigl et al. (5, 16). Each circle represents an allelic profile based on allele calling of 4,221 target genes. The numbers on the connecting lines refer to the number of allele differences. Colors represent different sequence types.

Overall, 20 isolates with a genome size ranging from 7,024,618 (Bp14) to 7,456,996 (Bp27) bp were assembled. The mean GC content was 68.1% (67.95–68.30%) (Table 1). Chromosome 1 had, on average, 4.0 Mbp (3,906,615–4,263,701 bp) and chromosome 2 had 3.2 Mbp (3,100,937–3,243,075 bp). Additionally, a 132 kbp plasmid (61.84% GC; 129 genes) was identified in isolate Bp26 using MOB-suite (v3.1.9 [19]), which showed the highest similarity to plasmid 1 of *B. pseudomallei* strain TSV202 (accession number: CP009155.1), with 43% query coverage and 99.43% sequence identity. On average, 6,457 (6,228–6,737) genes were identified in total, comprising 6,260 (6,042–6,508) protein-coding genes, 77 (76–81) RNA genes, and 120 (98–162) pseudogenes. cgMLST analysis (5, 16) showed that all 20 isolates were genetically distinct, although 5 isolates were closely related (<10 allele differences in 4,221 target genes; Fig. 1).

**TABLE 1:** Characteristics, assembly quality parameters, and accession numbers of 20 *B. pseudomallei* isolates originating from Vietnam^*a*^

Isolate name	RefSeq	GC (%)	Genome size					No. of genes				Raw read input							
			Size (bp)	Contig no.	Size chromosome1 (bp)	Size chromosome2 (bp)	Plasmid (bp)	Total no. of genes	No. of protein-coding genes	No. of RNA genes	No. of pseudogenes	SRA (Nanopore)	SRA (Illumina)	No. of Nanopore reads	No. of Illumina reads	*N*_50_ (Nanopore raw read input)	Genome coverage (Nanopore)	Genome coverage (Illumina)	BioSample accession no.
Bp01	GCF_052858755.1	68.15	7,206,361	2	4,092,017	3,114,344	–	6,431	6,249	76	106	SRR30791284	SRR30791283	253,948	6,551,166	11,826.0	336.3	136.2	SAMN43911426
Bp02	GCF_052858685.1	68.06	7,275,229	2	4,091,997	3,183,232	–	6,544	6,356	77	111	SRR30791272	SRR30791261	156,974	7,038,462	19,468.0	245.3	144.9	SAMN43911427
Bp05	GCF_052858145.1	68.15	7,230,632	2	4,080,232	3,150,400	–	6,469	6,293	77	99	SRR30791203	SRR30791282	167,247	6,578,806	18,381.0	269.0	136.3	SAMN43911430
Bp06	GCF_052857935.1	68.19	7,159,108	2	4,009,207	3,149,901	–	6,370	6,195	77	98	SRR30791281	SRR30791280	385,072	5,886,782	10,345.0	407.4	123.2	SAMN43911431
Bp11	GCF_052857225.1	68.23	7,147,827	2	3,976,171	3,171,656	–	6,425	6,240	78	107	SRR30791268	SRR30791266	363,040	2,999,356	10,701.0	402.8	41.9	SAMN43911436
Bp13	GCF_052857015.1	68.26	7,072,326	2	3,951,380	3,120,946	–	6,291	6,103	77	111	SRR30791262	SRR30791258	199,585	3,463,130	17,267.0	319.6	48.9	SAMN43911438
Bp14	GCF_052856205.1	68.30	7,024,618	2	3,906,615	3,118,003	–	6,228	6,042	77	109	SRR30791257	SRR30791255	149,863	2,738,836	20,188.0	272.1	38.9	SAMN43911439
Bp15	GCF_052855985.1	68.21	7,130,256	2	4,007,637	3,122,619	–	6,386	6,204	77	105	SRR30791254	SRR30791253	192,937	6,789,608	17,805.0	314.5	142.7	SAMN43911440
Bp16	GCF_052855615.1	68.21	7,131,998	2	4,007,748	3,124,250	–	6,389	6,207	77	105	SRR30791252	SRR30791249	197,647	8,484,816	16,778.0	306.8	178.3	SAMN43911441
Bp17	GCF_052855605.1	68.19	7,185,644	2	4,038,557	3,147,087	–	6,465	6,282	81	102	SRR30791248	SRR30791247	144,106	6,263,564	18,384.0	227.6	130.6	SAMN43911442
Bp18	GCF_052855315.1	68.05	7,246,324	2	4,051,722	3,194,602	–	6,455	6,244	76	135	SRR30791246	SRR30791245	159,438	3,815,296	18,767.0	258.9	52.6	SAMN43911443
Bp19	GCF_052855305.1	68.02	7,283,335	2	4,088,868	3,194,467	–	6,508	6,296	76	136	SRR30791233	SRR30791241	170,264	3,152,550	18,349.0	281.6	43.2	SAMN43911444
Bp22	GCF_052854895.1	68.22	7,072,286	2	3,971,349	3,100,937	–	6,274	6,091	78	105	SRR30791233	SRR30791232	361,912	7,497,796	12,216.0	444.3	158.8	SAMN43911447
Bp23	GCF_052854885.1	68.05	7,300,111	2	4,087,561	3,212,550	–	6,582	6,376	77	129	SRR30791231	SRR30791229	143,397	3,662,942	20,008.0	244.5	50.1	SAMN43911448
Bp24	GCF_052854615.1	68.10	7,226,221	2	4,051,444	3,174,777	–	6,480	6,276	77	127	SRR30791227	SRR30791225	252,633	7,152,088	10,807.0	282.1	148.2	SAMN43911449
Bp25	GCF_052854605.1	68.10	7,226,306	2	4,051,624	3,174,682	–	6,485	6,280	77	128	SRR30791224	SRR30791222	276,308	7,174,654	13,778.0	373.7	148.7	SAMN43911450
Bp26	GCF_052854305.1	68.03	7,337,923	3	4,063,971	3,142,130	131,822	6,582	6,343	77	162	SRR30791221	SRR30791220	157,631	8,308,968	18,521.0	262.5	169.6	SAMN43911451
Bp27	GCF_052854315.1	67.95	7,456,996	2	4,263,701	3,193,295	–	6,737	6,508	79	150	SRR30791219	SRR30791215	134,057	3,067,668	19,980.0	220.8	41.1	SAMN43911452
Bp29	GCF_052854015.1	68.04	7,337,715	2	4,142,467	3,195,248	–	6,557	6,348	76	133	SRR30791210	SRR30791209	210,287	7,457,826	16,531.0	306.8	152.3	SAMN43911454
Bp30	GCF_052854005.1	68.01	7,301,610	2	4,058,535	3,243,075	–	6,489	6,265	77	147	SRR30791208	SRR30791204	128,515	3,439,576	20,500.0	229.6	47.0	SAMN43911455

^*a*^ – indicates no plasmids detected.

## Data Availability

The assembled genomes have been deposited in NCBI GenBank and SRA (BioProject PRJNA1161571). Individual BioSample accession numbers for each genome are listed in Table 1.
